# Exploring the genetic causal relationship between physical activity and migraine in European population based on Mendelian randomization analysis

**DOI:** 10.3389/fneur.2024.1434433

**Published:** 2024-08-19

**Authors:** Jinfu Wang, Guan Yang

**Affiliations:** School of Physical Education, South China University of Technology, Guangzhou, Guangdong, China

**Keywords:** physical activity, migraine, Mendelian randomization, genetic epidemiology, causal relationship

## Abstract

**Background:**

Previous studies have shown a connection between physical activity and migraines, but they don’t prove a cause-and-effect relationship due to potential biases in observational methods.

**Methods:**

Utilizing accelerometer-measured physical activity data from a cohort of 377,234 participants in the UK Biobank and information from 599,356 European migraine patients (including 48,975 cases and 550,381 controls) obtained from 24 cohorts, we performed a bidirectional Mendelian randomization analysis to investigate the genetic bidirectional causal relationship between accelerometer-measured physical activity and migraines.

**Results:**

Research findings indicated a slight negative genetic correlation between “average acceleration” physical activity (*r_g_* = −0.091, *p* = 0.011), overall physical activity (*r_g_* = −0.081, *p* = 0.017), and migraine. Nevertheless, no shared genetic components were observed between migraine and “fraction of accelerations > 425 mg” of physical activity (*r_g_* = −0.124, *p* = 0.076). The study results also demonstrated a lack of genetic bidirectional causality between accelerometer-measured physical activity and migraine (“average acceleration”, *OR* = 1.002, 95% CI 0.975–1.031, *p* = 0.855, “fraction of accelerations > 425 mg”, *OR* = 1.127, 95% CI 0.802–1.583, *p* = 0.488, overall physical activity, *OR* = 0.961, 95% CI 0.713–1.296, *p* = 0.799), and vice versa. Additionally, this lack of causal association persists even after adjusting for obesity (*OR* = 1.005, *p* = 0.578), education (*OR* = 1.019, *p* = 0.143), and depression (*OR* = 1.005, *p* = 0.847), either separately or simultaneously.

**Conclusion:**

The Mendelian randomization results based on genetic data do not provide support for a causal association between physical activity and migraine.

## 1 Introduction

Migraine represents a prevailing and recurring neurological ailment (1), distinguished by episodic, pulsating headaches on one side, frequently accompanied by symptoms like nausea, vomiting, photophobia, or a confluence of these manifestations (2). Migraine is a widely acknowledged global health concern, standing as the foremost cause of disability worldwide (3). In 2016, the condition affected an estimated 1.05 billion individuals, resulting in over 45 million disability-adjusted life years and imposing substantial economic and social burdens on individuals and families (4). Moreover, migraine has been linked to a heightened risk of ischemic stroke (5) and cognitive impairment (6).

Various primary care approaches have been suggested for migraine treatment, encompassing acute and preventive medications, as well as lifestyle modifications (7, 8). According to guidelines, medication represents the principal treatment method for migraines (9). However, medication therapy has been associated with challenges, including adverse events (10), medication nonadherence (11), and medication overuse (12). Studies indicate that medication overuse escalates the frequency and intensity of headaches, creating a vicious cycle of drug dependency and headache attacks (12), underscoring the considerable obstacles in its application. Conversely, non-pharmacological treatments, such as physical activity, present a promising avenue for migraine treatment.

Several epidemiological studies have consistently revealed a robust association between increased physical activity and a diminished risk of experiencing migraines (13–15). Moreover, these studies have demonstrated a heightened prevalence of migraines among populations with lower levels of physical activity. Clinical trials further support the link between increased physical activity and migraines, consistently showcasing positive neurological or cardiovascular effects in migraine sufferers. These benefits include heightened nitric oxide levels (16), elevated concentrations of beta-endorphins, and the induction of endogenous cannabinoid release (17). However, while observational studies play a crucial role in establishing the connection between physical activity and migraines, their inherent limitations make them susceptible to confounding effects (e.g., physical activity might be linked to other factors affecting migraine) and reverse causation (e.g., a migraine attack could lead to reduced levels of physical activity). Additionally, existing observational studies have predominantly utilized self-report assessments of physical activity, which could yield biased results due to participant recall and social expectations. To address these limitations, device measures of physical activity are essential for assessing its genetic causal relationship with migraine.

Mendelian randomization (MR) is a statistical method that estimates the genetic causal relationship between exposure and outcomes by using germline genetic variations as instrumental variables. This approach provides more robust causal inferences compared to traditional observational studies because genetic variation is randomly assigned to offspring during meiosis at conception, thereby mitigating confounder effects and reverse causation. In this study, we aimed to evaluate if the apparent link between physical activity and migraines could be elucidated through a shared genetic foundation, utilizing genetic correlation analyses. Following this, a bidirectional Mendelian randomization analysis was performed to explore the genetic causal association between physical activity and migraines. Drawing on correlations documented in observational studies, we postulated the presence of genetic correlations between physical activity and migraines. Additionally, we hypothesize a bidirectional genetic causal relationship between physical activity and migraines.

## 2 Materials and methods

### 2.1 Overall study design

We performed a bidirectional, two-sample MR analysis to evaluate the genetic causal relationship between physical activity and migraine. The study design consisted of four key elements: (1) obtaining summary-level GWAS data for the exposure factors and outcome variables; (2) identifying genetic variants associated with exposure factors and outcome factors as instrumental variables (IVs); (3) employing a combination of MR analysis methods to ascertain causality between the exposure factors and the outcome variables; and (4) interchanging the exposure factors and the outcome variables in reverse MR analyses to ascertain the potential existence of a reverse causal relationship between them. In this study, the initial assumption was safeguarded by extracting genetic variants from GWAS summary statistics that exhibited genome-wide significance (*p* < 5×10^−8^) and a strong correlation with the exposure variable. Potential threats to the second and third assumptions encompass horizontal pleiotropy, linkage disequilibrium (LD) among genetic loci, and population stratification (18). To mitigate the influence of population stratification, we rigorously limited our analysis to individuals of European ancestry. Additionally, we employed the PLINK clustering method to alleviate the impact of LD between genetic variants. Multiple sensitivity analyses were conducted to scrutinize the presence of horizontal pleiotropy. Nevertheless, assessing the third assumption proves challenging when reliant on summary statistics rather than individual-level data.

### 2.2 Summary-level data on physical activity

The current study acquired summary datasets of physical activity from two recent GWAS conducted in the UK Biobank population.^1^ The UK Biobank is a large-scale population-based prospective cohort study encompassing nearly half a million adults aged 40–69 years and residing in the UK, providing extensive phenotypic and genotypic details of participants. To objectively assess physical activity, we selected two physical activity phenotypes from the GWAS of Klimentidis and colleagues on July 5, 2023: accelerometer-measured “average acceleration” physical activity and “fraction of accelerations >425 mg” physical activity, corresponding to levels of vigorous physical activity (6MET) (19). For the replication analysis, we further examined another GWAS pooled dataset, which measured overall activity levels using wristband accelerometers (20) (Table 1). These GWAS summary data for physical activity have been validated in previous studies (25). In these GWAS datasets, participants were instructed to wear wrist accelerometers continuously for a minimum of 72 h. Exclusions applied to individuals with insufficient data for less than 3 days (72 h) or no recorded data within each hour of a 24-h period. Adjustments for covariates in GWAS results included genotyping array, age squared, and wear season. The effect size was construed as the change in physical activity standard deviation per additional risk allele, approximately corresponding to substituting sedentary behavior with around 75 min of moderate activity (i.e., brisk walking) per day (19, 20). As we are utilizing summary-level data, we will refer to the original preliminary version to obtain relevant information.

**Table 1:** MR study summary of GWAS data.

Phenotype	Author and year	Sample size	Ancestry	Main consortium	PubMed ID
Physical activity	Klimentidis et al. (19)	91,084	European	UK Biobank	29,899,525
Physical activity	Doherty et al. (20)	91,105	European	UK Biobank	30,531,941
Migraine	Hautakangas et al. (21)	599,356	European	IHGC	35,115,687
Obesity	Locke et al. (22)	339,224	European	UK Biobank	25,673,413
Education	Schoeler et al. (23)	283,749	European	UK Biobank	37,106,081
Depression	Wray et al. (24)	480,359	European	UK Biobank	29,700,475

IHGC, the International Headache Genetics Consortium.

### 2.3 Summary-level data on migraine

The summary genetic statistics associated with migraine were retrieved from the largest recent meta-analysis of GWAS, which amalgamated data from 873,341 samples of European ancestry sourced from five study collections. Of these, the assessment of participants’ migraines is conducted by physicians based on the second edition of the International Classification of Headache Disorders or through self-reporting by participants. This extensive GWAS meta-analysis identified 123 risk loci (*p* < 5 × 10^−8^) significantly associated with migraine (including data from the 23andMe cohort) (21). However, the 23andMe cohort discovery dataset was excluded from the study to safeguard the privacy of the 23andMe cohort study participants. Consequently, the summary dataset for migraine in this investigation originated from 599,356 European-origin samples (48,975 cases and 550,381 non-cases) obtained from the remaining 24 cohort studies (Table 1) (accessed on 7 July 2023) (see Supplementary Tables S1, S2 for detailed cohort characteristics). Given that we employed summary-level data as opposed to individual-level data, a comprehensive presentation of descriptive statistics and details regarding sample size limitations cannot be furnished. Instead, all acquired information is extracted from the original publications. Therefore, this study involves a secondary analysis of existing data, classifying it as a *post hoc* analysis.

### 2.4 Genetic correlation analysis

To assess the genetic correlation between objectively measured physical activity and migraine, we performed cross-trait linkage disequilibrium score regression (LDSC) with default parameters. While considering linkage disequilibrium between SNPs, we employed aggregate statistics to quantify the genomic genetic overlap. By adopting the Bonferroni-corrected *p*-values (*p* < 0.016, 0.05/3) as the threshold of significance, *p*-values falling within the range of 0.016–0.05 were indicative of suggestive associations.

### 2.5 Genetic instruments selections

**Figure 1:**
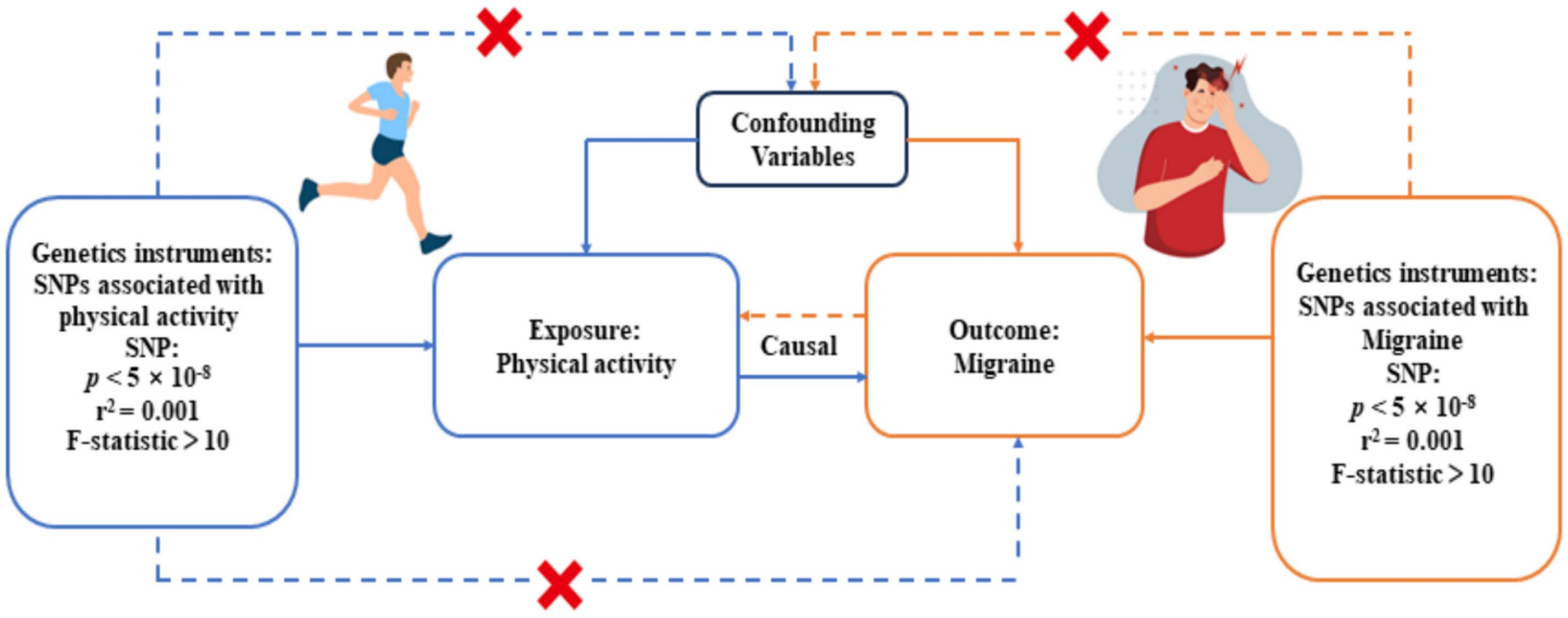
Study design overview. SNP, Single nucleotide polymorphism.

In this MR analysis, to adhere to the assumptions of MR analysis, we employed a significance threshold of *p* < 5 × 10^−8^ to select Single Nucleotide Polymorphisms (SNPs) with statistical significance from the GWAS summary data as the preliminary set of instrumental variables to ensure that the selected SNPs have a highly significant and reliable association with the exposure factors. To further screen for independent SNPs, we applied the PLINK clustering method with specific parameters (*r^2^* = 0.001, cluster distance = 10,000 kb), chosen to ensure genetic independence while maintaining adequate representation of the selected SNPs. In cases where specific exposure-associated SNPs were absent in the resultant dataset, we replaced them with LD proxies (*r^2^* > 0.8) to ensure that genetic variations associated with the exposure factors were included in the analysis. If the number of instrumental variables identified was less than 3 using these criteria, we adopted a more lenient threshold *p*-value (*p* < 5 × 10^−7^) to identify sufficient instrumental variables, a practice commonly used in other MR studies (26). Moreover, to minimize the bias of weak instruments, we only considered SNPs with F-statistics >10 as potential instrumental variables, an important metric for measuring the strength of instrumental variables, where a higher *F*-value indicates a stronger association between SNPs and exposure factors. In the final analysis, a total of 15 IVs for objectively measured physical activity and 35 IVs for migraine-associated variables were included. For more detailed information on these IVs, refer to Supplementary Tables S3–S6. The flowchart of our MR study design is presented in Figure 1.

### 2.6 Two sample MR analysis

In this study, we conducted a bidirectional two-sample MR study using the TwoSample-MR 3.2R package developed by researchers on the MR-base platform (27). Positive MR analyses were performed, with objectively measured physical activity as the exposure and migraine as the outcome. Conversely, reverse MR analysis was conducted, with migraine as the exposure and objectively measured physical activity as the outcome.

We employed the IVW approach to evaluate the causal association between exposure factors and outcome variables (28). The IVW method assigns weights to each SNP that are inversely proportional to its variance, taking into account the statistical power of each SNP. The advantage of this method lies in its ability to integrate information from various genetic instruments, thereby enhancing the overall statistical power of the analysis. In cases of heterogeneity (*Q*-value <0.05), we used a multiplicative random-effects IVW model; otherwise, a fixed-effects IVW model was used (29). The method is widely utilized in MR analyses due to its statistical efficacy and reliability of causal estimates while allowing each SNP to have distinct average effects (28). However, it assumes that all SNPs are valid, and that no horizontal pleiotropy exists, if horizontal pleiotropy is present (where the instrumental variable influences the outcome through a causal pathway other than the exposure), the effect estimates may be biased. Therefore, we further use four other commonly used MR complementary methods (weighted median method, weighted mode method, MR-Egger method, and simple mode method) to validate the reliability of the main results, even though these methods come at the cost of reduced statistical efficacy (30).

To control for the risk of false positives associated with multiple testing, we applied the Bonferroni correction. This technique adjusts the *p*-value by dividing the traditional significance threshold of 0.05 by the number of independent tests performed. In this study, we conducted tests on three exposure factors and one outcome variable, with each pair undergoing both forward and reverse MR analyses, resulting in six tests in total. Therefore, a *p*-value less than 0.008 was used to determine statistical significance for the causal inferences. No statistical power calculations were performed before the study as only previously collected GWAS summary statistics were used. Information on sample size and other details is based on existing original publications. All significance testing was two-tailed.

### 2.7 Confounding factors

To investigate potential mechanisms influencing genetic associations between exposure factors and outcome variables, we conducted multivariable MR analyses and adjusted for several potential risk factors that could introduce relevant pleiotropy (31). Prior meta-analyses and MR studies have indicated associations of instrumental variables with traits related to obesity, education, and depression (8, 26, 32, 33). Consequently, we selected SNPs associated with obesity from a GWAS comprising 339,224 individuals (22) and SNPs related to education (defined by the International Standard Classification of Education 2011) from a GWAS involving 283,749 individuals (23). For depression (defined by DSM-IV, the International Classification of Diseases, Ninth Revision, or the International Statistical Classification of Diseases and Related Health Problems, Tenth Revision), we employed pooled GWAS data from 480,359 individuals in the UK Biobank population for the multivariable MR analysis (24) (Table 1). We utilized the inverse variance weighted (IVW) method to estimate the effect sizes, and the results were considered statistically significant when the two-tailed *p* < 0.001 using Bonferroni to corrected values (0.008/3/2, with 3 denoting Bonferroni corrected three tests: three exposures and one outcome, and 2 denoting two directions of MR analysis).

### 2.8 Sensitivity analysis

To validate the reliability of the results, we conducted comprehensive sensitivity analyses, including assessments for heterogeneity and pleiotropy. Firstly, employing the Cochran Q statistic to measure heterogeneity, which is a widely used measure in the field (9). Heterogeneity was considered to be present when the Q statistic yielded a large value with a corresponding *p* < 0.05, and this suggested that SNPs associated with individual exposure phenotypes might exert substantial influence on the results, necessitating careful interpretation. Secondly, MR-Egger regression analysis was employed to assess the presence of horizontal pleiotropy, wherein a significant intercept (*p* < 0.05) indicates the existence of pleiotropic effects. Moreover, in the context of IVW linear regression, we employed MR-PRESSO analysis to identify probable outlier instrumental variables and generate IVW estimates after removing these outliers. Furthermore, we utilized the *p*-value of the MR-PRESSO distortion test to determine whether the estimates significantly differed before and after correction for the outlier instrumental variables. Finally, we performed Loo analyses to assess whether the pooled estimates were driven or biased by individual SNPs. All significance testing was two-tailed.

## 3 Results

### 3.1 Genetic correlations

As per the LDSC results (Table 2), we have unveiled a suggestive negative genetic correlation between the “average acceleration” physical activity (*r_g_* = −0.091, *p* = 0.011), overall physical activity measured (*r_g_* = −0.081, *p* = 0.017), and migraine. Nevertheless, no shared genetic components were observed between migraine and “fraction of accelerations >425 mg” physical activity (*r_g_* = −0.124, *p* = 0.076).

**Table 2:** Genetic correlations between objectively measured physical activity and migraine.

Phenotype 1	Phenotype 2	*R_g_*	SE	*Z*	*p*
Average acceleration	Migraine	−0.091	0.031	−2.612	0.011
Fraction of accelerations >425 mg	Migraine	−0.124	0.074	−1.790	0.076
Overall physical activity	Migraine	−0.081	0.032	−2.401	0.017

R_g_, genetic correlations.

### 3.2 The causal effects of physical activity on migraine

**Figure 2:**
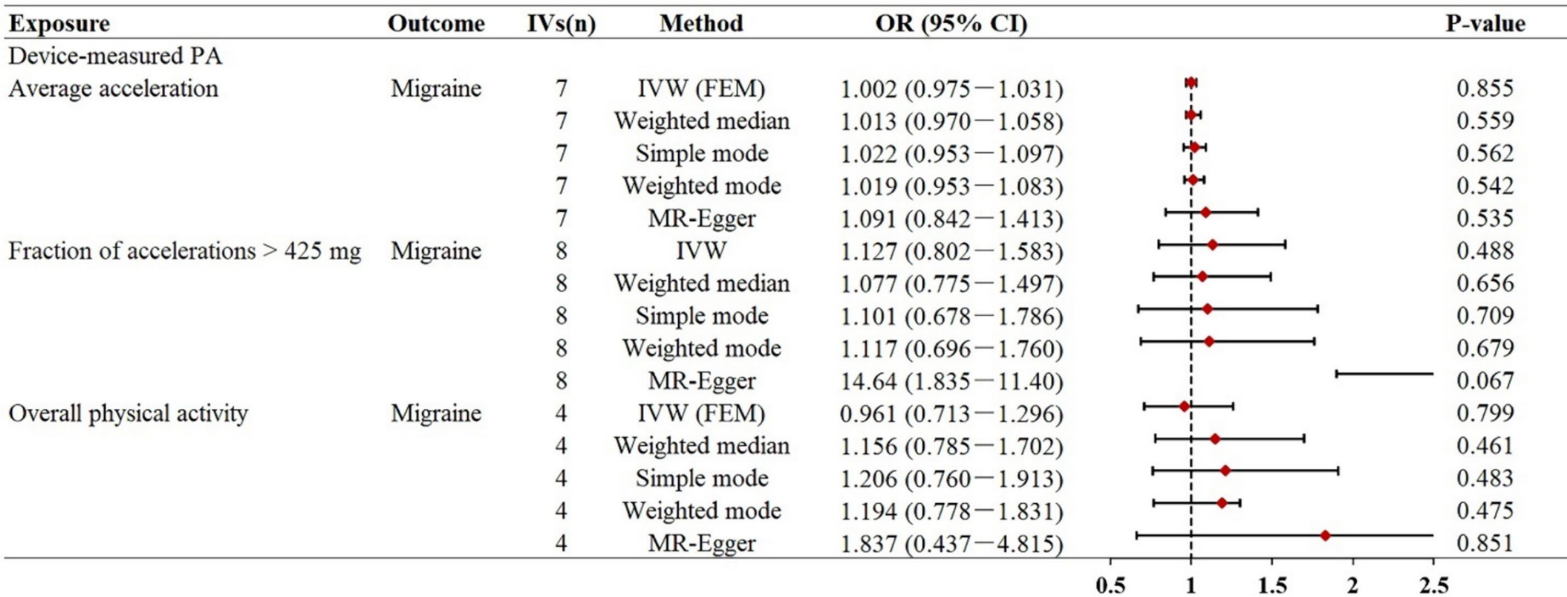
Mendelian randomization estimates for the relationship between accelerometer-based physical activity and migraine. Summary of Mendelian randomization estimates obtained from inverse variance weighting, weighted median, simple mode, weighted mode, and MR-egger method. CI, confidence interval; FEM, fixed effects model; MR, Mendelian randomization; IVs, instrumental variables; OR, odds ratio; IVW, inverse variance weighted.

In this direction, based on the results of the IVW MR analysis, our study did not reveal a genetic causal association between objectively measured physical activity and migraine (Figure 2). The effect coefficients for both phenotypes were estimated as 1.002 (*p* = 0.855) and 1.127 (*p* = 0.488), suggesting that physical activity likely has no significant impact on migraine. Moreover, the other four MR supplementation methods consistently indicated no genetic causal link between physical activity and migraine. In our replication analysis, the results consistently demonstrated that there was no direct genetic causal relationship between overall physical activity measured by accelerometers and the risk of migraines, whether assessed using the IVW method (*OR* = 0.961, *p* = 0.799) or other sensitivity analyses. Scatter plots between physical activity and migraine risk can be found in Supplementary Figures S1–S3.

### 3.3 The causal effects of migraine on physical activity

**Figure 3:**
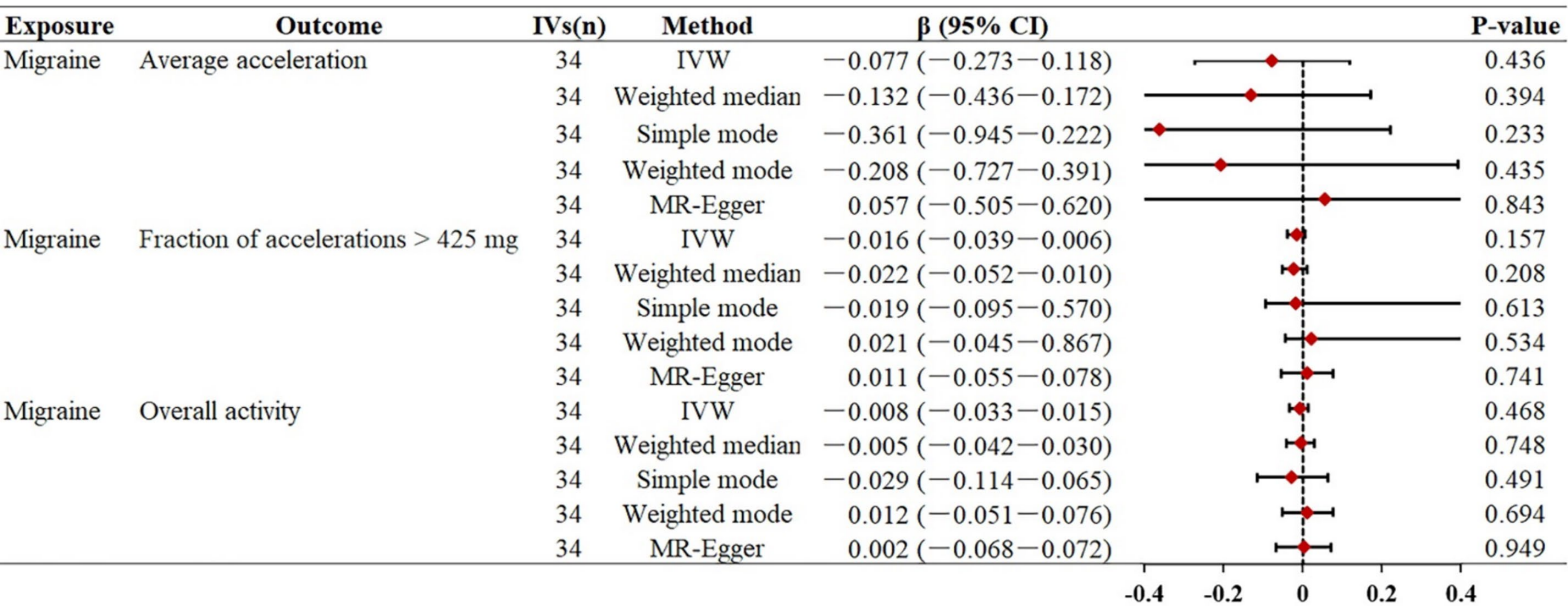
Mendelian randomization estimates for the relationship between migraine and physical activity. Summary of Mendelian randomization estimates obtained from inverse variance weighting, weighted median, simple mode, weighted mode, and MR-egger method. CI, confidence interval; FEM, fixed effects model; MR, Mendelian randomization; IVs, instrumental variables; OR, odds ratio; IVW, inverse variance weighted.

Similar to previous analyses, the primary IVW analysis did not yield strong evidence supporting a genetic causal relationship between migraine and objectively measured physical activity (Figure 3). In our primary analysis, migraine represented by 34 SNPs was not statistically associated with accelerometer-measured “average acceleration” (*β* = −0.077, *p* = 0.436), “fraction of accelerations >425 mg” (*β* = −0.016, *p* = 0.157), and overall physical activity (*β* = −0.008, *p* = 0.468), suggesting that migraine may have no significant effect on objectively measured physical activity. Consistently, the other four MR supplementation methods produced similar effects. Scatter plots between migraine and physical activity can be found in Supplementary Figures S4, S5.

### 3.4 Multivariable MR analysis

To further investigate potential risk factors that might impact the causal association between objectively measured physical activity and migraine, we performed multivariable MR analyses by adjusting separately or simultaneously for the variables of obesity, education, and depression. Our findings indicated that there was no statistically significant association between accelerometer-measured physical activity and migraine (“average acceleration,” *p =* 0.738, “fraction of accelerations >425 mg,” *p =* 0.294), and vice versa (Tables 3, 4).

**Table 3:** Multivariable MR results of objectively measured physical activity on risk of migraine.

Exposure	Outcome	Model	*OR* (95% CI)	*p-*value
Average acceleration	Migraine	IVW adjusted for obesity	1.000 (0.981–1.022)	0.578
		IVW adjusted for education	1.010 (0.992–1.043)	0.143
		IVW adjusted for depression	1.000 (0.954–1.053)	0.847
		IVW fully adjusted model	0.991 (0.971–1.013)	0.738
Fraction of accelerations >425 mg	Migraine	IVW adjusted for obesity	0.992 (0.842–1.184)	0.991
		IVW adjusted for education	1.141 (0.862–1.501)	0.334
		IVW adjusted for depression	0.710 (0.46–1.103)	0.136
		IVW fully adjusted model	0.872 (0.684–1.124)	0.294
Overall physical activity	Migraine	IVW adjusted for obesity	1.031 (0.891–1.193)	0.685
		IVW adjusted for education	1.032 (0.832–1.281)	0.764
		IVW adjusted for depression	0.623 (0.374–1.033)	0.065
		IVW fully adjusted model	0.953 (0.781–1.152)	0.634

IVW, inverse-variance weighted.

**Table 4:** Multivariable MR results of migraine on objectively measured physical activity.

Exposure	Outcome	Model	*β* (95% CI)	*p-*value
Migraine	Average acceleration	IVW adjusted for obesity	0.094 (−0.274–0.462)	0.617
		IVW adjusted for education	−0.010 (−0.284–0.263)	0.941
		IVW adjusted for depression	−0.022 (−0.234–0.188)	0.833
		IVW fully adjusted model	−0.119 (−0.543–0.304)	0.581
Migraine	Fraction of accelerations >425 mg	IVW adjusted for obesity	0.008 (−0.029–0.046)	0.673
		IVW adjusted for education	0.015 (−0.012–0.043)	0.288
		IVW adjusted for depression	0.007 (−0.018–0.033)	0.579
		IVW fully adjusted model	−0.005 (−0.049–0.038)	0.813
Migraine	Overall physical activity	IVW adjusted for obesity	0.007 (−0.037–0.052)	0.749
		IVW adjusted for education	−0.002 (−0.036–0.031)	0.896
		IVW adjusted for depression	−0.004 (−0.031–0.021)	0.723
		IVW fully adjusted model	−0.039 (−0.632–0.553)	0.896

IVW, inverse-variance weighted.

### 3.5 Sensitivity analysis

The heterogeneity and horizontal pleiotropy among genetic instruments are presented in Table 5. MR-Egger regression showed that there was no significant horizontal pleiotropy in the association between physical activity and migraine (“average acceleration” intercept *p* = 0.534, “fraction of accelerations >425 mg” intercept *p* = 0.071, and overall physical activity intercept *p* = 0.839), and vice versa. Loo analyses showed that causal estimation was not driven or biased by any individual SNPs driven or biased (see Supplementary Figures S7–S12), implying that our results are robust and valid. To filter SNPs instrumented with migraine outcomes that may exhibit polymorphic associations, we further used the MR-PRESSO global test to exclude potentially pleiotropic SNPs. Specifically, rs11012732, rs34517439, and rs6775319 were excluded from the “average acceleration” physical activity analysis; rs6775319 was excluded from the overall physical activity analysis. After removing these outliers, despite eliminating heterogeneity, we found no difference in the estimates compared with those before removal (*P* for MR-PRESSO distortion tests >0.05). Using the remaining SNPs as IV, the results showed that objectively measured physical activity was not associated with migraine risk (see Supplementary Figure S13). In addition, sensitivity analyses showed that the other four supplementary methods of MR analysis all provided similar effects to the main analysis. Scatter plots of the remaining IVs associated with migraine and the results of the Loo analysis can be found in Supplementary Figures S14–S17. In our present study, the F-statistic for IV ranged from 20.26 to 4,055, indicating a low probability of weak instrumental bias affecting our MR analysis. Furthermore, there was only a small sample overlap between the GWAS data for physical activity and migraine (absolute deviation <0.005), suggesting that bias from sample overlap is unlikely to be a concern (see Supplementary Figures S18–S23).

**Table 5:** Sensitivity analysis.

Exposure	Outcome	MR-egger regression		Cochran’s *Q*-test	
		ERI	*p-*value	*Q*	*Q* value
Average acceleration	Migraine	−0.021	0.534	28.60	7.23 × 10^−5^
Fraction of accelerations >425 mg	Migraine	−0.123	0.071	13.65	5.76 × 10^−2^
Overall activity	Migraine	−0.011	0.839	12.68	5.37 × 10^−3^
Migraine	Average acceleration	−0.012	0.619	30.47	5.93 × 10^−1^
Migraine	Fraction of accelerations >425 mg	−0.033	0.386	35.91	3.33 × 10^−1^
Migraine	Overall activity	−0.054	0.741	33.55	4.40 × 10^−1^

ERI, egger regression intercept.

## 4 Discussion

In this study, our findings provide crucial evidence that there is no genetic causal relationship between objectively measured physical activity and migraine. Likewise, migraine does not demonstrate a genetic causal association with reduced physical activity. Moreover, this absence of association remains consistent across our replication analyses. To our knowledge, this is the pioneering study employing MR analysis to evaluate the causal link between objectively measured physical activity and migraine. However, it is important to note that the elements that precipitate or protect against a condition may not correspond to those that influence the progression of an established illness. The findings we present are not counter-indicative of the potential role of physical activity in mitigating the progression of the disease in those already identified with migraines. Consequently, the ongoing trials involving physical activity as an intervention for migraine patients should not be inherently impacted by the conclusions drawn from our study.

Our findings are consistent with several previous epidemiological studies that have found no association between physical activity and migraine. For instance, a cross-sectional study carried out in Denmark found no evident correlation between the level of physical exercise and the prevalence of migraines (*OR* = 0.501, *p* = 0.121) (34). A randomized controlled trial involving 37 individuals with migraines suggested that engaging in physical exercise did not significantly impact the frequency of migraine days (*SMD* = 1.052, *p* = 0.132) (35). Another randomized controlled trial also indicated that physical activity did not exert a notable influence on migraine symptoms among individuals with tension-type headaches and episodic migraines (−6 headache days per month, *p* = 0.098) (36). Additionally, a prospective cohort study illustrated that migraine patients engaging in moderate to vigorous exercise at least three times a week witnessed a reduction in monthly headache days; however, this reduction did not attain statistical significance (−0.4 headache days per month, *p* = 0.636) (37). Similarly, a clinical trial demonstrated that, despite enhancements in the frequency, intensity, and duration of migraines with physical exercise interventions, these improvements did not reach statistical significance (38).

However, the relationship between physical activity and migraine risk remains inconsistent in previous observational studies. Most observational studies indicate a protective effect of physical activity on migraine development. For instance, in analyzing baseline data from the Brazilian Longitudinal Cohort Study of Adult Health, Oliveira et al. found that adults with the highest levels of self-reported leisure time physical activity had a 29% decreased risk of migraine without aura compared with inactive adults (39). In addition, a cohort study conducted in Canada reported that women in the physical activity group had a 14% reduction in migraine risk after adjusting for social/health factors (40). Notably, both cohort studies were conducted in non-European populations.

The difference in outcomes between the prospective cohort studies and the MR analyses can be explained in several ways. On the one hand, it is worth noting that both observational studies were conducted in non-European populations, whereas our MR analysis population consisted solely of individuals of European ancestry. This demographic contrast could potentially lead to variations in the clinical presentation and diagnostic modalities of migraine between non-European and European populations. On the other hand, despite the prospective cohort studies’ efforts to control for potential confounders affecting the relationship between physical activity and migraine, such as extending the follow-up time (lag) between physical activity assessment and migraine diagnosis, the possibility remains that this relationship may manifest early in the course of migraine disease (15). Moreover, there may be potential selection bias. We note that most of these cohort studies relied on how the questionnaires were administered, which may have led less motivated individuals to forgo completing the questionnaires, and thus, these results may underestimate the observed relationship.

Although our forward MR analysis suggests that physical activity does not have a significant impact on migraine, it may have a positive impact on migraine sufferers and the resulting economic burden. Previous research has revealed that physical activity can reduce the frequency, duration, intensity, and associated disability of migraine (38). Physical activity can also improve migraine-related quality of life and confer multiple health benefits on patients, for instance, sleep regulation, weight management, and improved psychological well-being (41–43). Additionally, physical activity can boost an individual’s self-efficacy and thus expectations for future outcomes (41), and research has demonstrated that individuals with high self-efficacy are more confident and capable of preventing migraine attacks or managing the burdens associated with migraine (44). More importantly, physical activity typically carries a lower risk of side effects and does not carry a significant cost burden. Thus, given the combination of low cost, low risk, and health benefits, it may be feasible to encourage physical activity in people with.

Our MR study possesses significant strengths. Firstly, a previous MR study indicated a correlation between subjectively measured light physical activity and migraine (9). However, previous research has indicated that subjectively measured physical activity tends to overestimate the actual time individuals engage in physical activity (45). In contrast, we assessed the causal association between objectively measured physical activity and migraine, which is more precise and reliable than subjectively measured data. Secondly, we comprehensively assessed the association between physical activity and migraine by analyzing three types of physical activity: “average acceleration” measured by accelerometers, “fraction of accelerations >425 mg,” and overall physical activity.

Several limitations of our MR study should be acknowledged. Firstly, despite obtaining instrumental variables from the largest available GWAS data on objective measures of physical activity, only a few of the instrumental variables were significant. Consequently, caution is advised in the interpretation of causal implications within the present study (46), given the potential for inadequate statistical power in the MR analysis. Secondly, our GWAS data are primarily derived from individuals of European ancestry. While this approach helps minimize bias arising from population stratification, we acknowledge that there is a possibility that our study findings may not be fully generalizable to other populations with distinct genetic backgrounds, such as Asian individuals. Consequently, it is essential to replicate our study in diverse populations. Thirdly, since we utilized summary-level data in our MR analysis, we cannot rule out the possibility of a non-linear causal relationship between objectively measured physical activity and the risk of migraines. Future studies should assess the potential dose–response causal relationship between physical activity and the risk of migraines in further MR studies using individual-level data and longitudinal designs. Additionally, Migraine outcomes may be subject to some degree of misclassification bias, as the assessment of migraines by the International Headache Society involves not only physician diagnosis but also self-reporting by investigators, introducing potential biases. The self-reported migraine prevalence from the International Society (excluding the 23andMe cohort) is 8.2%, falling within the range of migraine prevalence in Europe (6.4–11.7%) (47), we recommend that future studies employ more precise diagnostic methodologies to enhance the accuracy of the results. Ultimately, the specific demographic representation within our dataset may limit our comprehension of the global prevalence of migraine. Consequently, we advocate for the inclusion of a more diverse sample in the design of future studies to assess the global prevalence of migraine and its potential association with physical activity more thoroughly.

## 5 Conclusion

In summary, the present MR study provides genetic evidence endorsing the lack of a bidirectional causal link between physical activity and migraines. Our research findings suggest that the available evidence supporting physical activity as a preventive measure for migraines is currently insufficient. Prior findings concerning the association between physical activity and migraine risk could be affected by confounding factors. However, despite physical activity not being a direct preventive for migraines, its role in enhancing overall health warrants promotion in public health advice. Future research should involve broader populations to confirm our findings and explore the potential benefits of physical activity in managing migraine symptoms, utilizing more precise diagnostics and individual-level data for a detailed understanding of their interplay. The findings urge a reassessment of migraine guidance, ensuring it is based on robust scientific evidence. Further experimental research is essential to corroborate our results, enhance the understanding of the relationship between physical activity and migraines, and refine management strategies for those affected.

## Data Availability

The original contributions presented in the study are included in the article/Supplementary material, further inquiries can be directed to the corresponding author.

## Footnotes

^1^ https://www.ebi.ac.uk/gwas/
