# Supplementary material for: Exploring the genetic causal relationship between physical activity and migraine in European population based on Mendelian randomization analysis
Source: Front Neurol. 2024 Aug 19;15:1434433. doi: 10.3389/fneur.2024.1434433 (PMC11367984; doi:10.3389/fneur.2024.1434433)
Supplement: Supplementary file 1 [file Data_Sheet_1.docx]

Exploring the Genetic Causal Relationship between Physical Activity and Migraine in European Population Based on Mendelian Randomization Analysis

**1 Supplementary Tables and Figures**

**1.1 Supplementary Tables**

**Table S1.** Detailed study cohort characteristics.

| Abbreviation | Full Name | Study Design | Control Samples | Ancestry | Cases | Controls | Case % | Migraine Definition | Control Definition |
| --- | --- | --- | --- | --- | --- | --- | --- | --- | --- |
| IHGC2016* | Gormley et al. (no 23andMe) | Population and clinic | Within cohort | European descent | 29,209 | 172,931 | 14.4 | Self-reported and ICHD-II | No migraine; population |
| UKBB | UK Biobank | Population | Within cohort | European, British | 10,881 | 330,170 | 3.2 | Self-reported | No migraine, no headache; population |
| GeneRISK | GeneRISK Study | Population | Within cohort | European, Finnish | 1,084 | 4,857 | 18.2 | Self-reported | No migraine |
| HUNT | Nord-Trøndelag Health Study | Population | Within cohort | European, Norwegian | 7,801 | 32,423 | 19.4 | Self-reported migraine or fulfilling modified ICHD-II criteria | No headache, population |

**Table S2.** All 21 cohorts included in the IHGC2016 from Gormley et al. 2016 (no23andMe)

| Abbreviation | Full Name | Study Design | Control Samples | Ancestry | Cases | Controls | Case % | Migraine Definition | Control Definition |
| --- | --- | --- | --- | --- | --- | --- | --- | --- | --- |
| ALSPAC | Avon Longitudinal Study of Parents and Children | Population | Within cohort | European, British | 3,134 | 5,103 | 38.0 | Self-reported migraine, current or prior | No migraine or use of migraine medications; population |
| ATM | Australian Twin Migraine | Population | Within cohort | European descent | 1,683 | 2,383 | 41.4 | Modified ICHD-II criteria, current migraine | Population |
| B5C | 1958 British Birth Cohort | Population | Within cohort | European, British | 1,165 | 4,141 | 22.0 | Self-reported migraine, current or prior | No migraine or severe recurrent headaches |
| Danish HC | Danish Headache Center | Clinic | Danish Blood Donor Study | European, Danish | 1,771 | 1,000 | 63.9 | ICHD-II | No migraine |
| DeCODE | deCODE Genetics Inc. | Population | Within cohort | European, Icelandic | 3,135 | 95,585 | 3.2 | Full ICHD-II criteria, current migraine | No migraine; population |
| Dutch MA | Dutch migraine with aura | Clinic | Rotterdam I | European, Dutch | 734 | 5,211 | 12.3 | ICHD-II | Population |
| Dutch MO | Dutch migraine without aura | Clinic | Rotterdam II | European, Dutch | 1,115 | 2,028 | 35.5 | ICHD-II | Population |
| EGCUT | Estonian Genome Center, University of Tartu | Population | Within cohort | European, Estonian | 813 | 9,850 | 7.6 | Self-reported | No migraine |
| Finnish MA | Finnish migraine with aura | Clinic | Helsinki Birth Cohort (1,697); FINRISK population controls (1,018) | European, Finnish | 933 | 2,715 | 25.6 | ICHD-II | Population; Population. |
| German MA | German migraine with aura | Clinic | Heinz Nixdorf Recall study (365); PopGen (645) | European, German | 1,071 | 1,010 | 51.5 | ICHD-II | Population; Population. |
| German MO | German migraine without aura | Clinic | KORA (801); GSK (846) | European, German | 1,160 | 1,647 | 41.3 | ICHD-II | Population; Population. |
| Health2000 | Health 2000 | Population | Within cohort | European, Finnish | 136 | 1,764 | 7.2 | Self-reported | Migraine-free population |
| HUNT | Nord-Trøndelag Health Study | Population | HUNT population controls; HUNT lung cancer; HUNT pre-eclampsia | European, Norwegian | 1,395 | 1,011 | 58.0 | Self-reported migraine or fulfilling modified ICHD-II criteria, current migraine | No migraine; population |
| NFBC | Northern Finnish Birth Cohort | Population | Within cohort | European, Finnish | 756 | 4,393 | 14.7 | Self-reported migraine, current or prior | No migraine |
| NTR/NESDA | Netherlands Twin Register and the Netherlands Study of Depression and Anxiety | Population | Within cohort | European, Dutch | 1,636 | 3,819 | 30.0 | Modified ICHD-II criteria, current migraine | No migraine or severe recurrent headache. |
| Rotterdam III | Rotterdam Study III | Population | Within cohort | European, Dutch | 487 | 2,175 | 18.3 | Modified ICHD-II criteria, current migraine | No migraine |
| Swedish Twins | Swedish Twins Registry | Population | Within cohort | European, Swedish | 1,307 | 4,182 | 23.8 | Self-reported or fulfilling modified ICHD-II criteria | No migraine |
| Tromsø | The Tromsø Study | Population | Within cohort | European, Norwegian | 660 | 2,407 | 21.5 | Self-reported or fulfilling modified ICHD-II criteria | No migraine |
| Twins UK | Twins UK | Population | Within cohort | European, British | 618 | 2,334 | 20.9 | Self-reported migraine or fulfilling Modified ICHD-II criteria, current or prior migraine | No migraine |
| WGHS | Women’s Genome Health Study | Population | Within cohort | European descent | 5,122 | 18,108 | 22.0 | Self-reported migraine or fulfilling Modified ICHD-II criteria, current or prior migraine | No migraine |
| Young Finns | Young Finns | Population | Within cohort | European, Finnish | 378 | 2,065 | 15.5 | Full ICHD-II criteria, current migraine | No migraine |

**Reference:**

Hautakangas, H., Winsvold, B. S., Ruotsalainen, S. E., et al. (2022). Genome-wide analysis of 102,084 migraine cases identifies 123 risk loci and subtype-specific risk alleles. *Nat. Genet.* 54, 152-160. doi: 10.1038/s41588-021-00990-0

Gormley, P., Anttila, V., Winsvold, B. S., et al. (2016). Meta-analysis of 375,000 individuals identifies 38 susceptibility loci for migraine. *Nat. Genet.* 48, 856-66. doi: 10.1038/ng.3598.

**Table S3.** Characteristics of 8 instruments strongly associated with “acceleration fraction > 425 mg” physical activity.

| SNP | A1 | A2 | EAF | Beta | SE | *P*-value | *F*-statistic |
| --- | --- | --- | --- | --- | --- | --- | --- |
| rs1668835 | T | A | 0.688 | -0.022 | 0.011 | 3.10E-07 | 20.266 |
| rs1856329 | A | C | 0.800 | 0.027 | 0.012 | 9.00E-08 | 21.845 |
| rs4754194 | C | T | 0.772 | -0.025 | 0.014 | 2.40E-07 | 20.564 |
| rs62443625 | T | C | 0.766 | -0.025 | 0.021 | 1.40E-07 | 21.295 |
| rs6433478 | T | C | 0.456 | -0.023 | 0.012 | 1.20E-08 | 25.317 |
| rs72633364 | G | A | 0.711 | -0.023 | 0.016 | 4.10E-07 | 20.467 |
| rs743580 | A | G | 0.519 | 0.024 | 0.004 | 1.30E-09 | 28.207 |
| rs80028338 | A | C | 0.794 | -0.027 | 0.005 | 1.50E-07 | 22.497 |

SNP: single nucleotide polymorphism; A1: effect allele; A2: other allele; EAF: effect allele frequency.

**Table S4.** Characteristics of 7 instruments strongly associated with “average acceleration” physical activity.

| SNP | A1 | A2 | EAF | Beta | SE | *P*-value | *F*-statistic |
| --- | --- | --- | --- | --- | --- | --- | --- |
| rs11012732 | A | G | 0.667 | 0.225 | 0.038 | 5.40E-09 | 2093.0 |
| rs12522261 | G | A | 0.656 | 0.211 | 0.048 | 3.90E-08 | 1858.1 |
| rs148193266 | A | C | 0.957 | -0.510 | 0.092 | 3.10E-08 | 1980.7 |
| rs34517439 | C | A | 0.879 | 0.307 | 0.066 | 4.40E-08 | 1874.4 |
| rs59499656 | A | T | 0.655 | -0.228 | 0.038 | 2.40E-09 | 2195.3 |
| rs6775319 | A | T | 0.271 | 0.225 | 0.041 | 3.50E-08 | 1859.7 |
| rs9293503 | T | C | 0.888 | 0.328 | 0.056 | 2.10E-08 | 1996.1 |

SNP: single nucleotide polymorphism; A1: effect allele; A2: other allele; EAF: effect allele frequency.

**Table S5.** Characteristics of 7 instruments strongly associated with overall physical activity.

| SNP | A1 | A2 | EAF | Beta | SE | *P*-value | *F*-statistic |
| --- | --- | --- | --- | --- | --- | --- | --- |
| rs11012732 | T | A | 0.655 | -0.028 | 0.004 | 1.90E-09 | 134.73 |
| rs59499656 | A | T | 0.271 | 0.037 | 0.004 | 3.90E-08 | 112.09 |
| rs6775319 | A | T | 0.663 | 0.036 | 0.004 | 2.30E-08 | 119.44 |
| rs9293503 | T | C | 0.888 | 0.041 | 0.007 | 4.90E-08 | 114.25 |

SNP: single nucleotide polymorphism; A1: effect allele; A2: other allele; EAF: effect allele frequency.

**Table S6.** Characteristics of 8 instruments strongly associated with migraine (Migraines to physical activity) (includes three phenotypes of physical activity).

| SNP | A1 | A2 | EAF | Beta | SE | *P*-value | *F*-statistic |
| --- | --- | --- | --- | --- | --- | --- | --- |
| rs10166942 | C | T | 0.196 | -0.114 | 0.009 | 5.89E-32 | 2501.2 |
| rs1019990 | T | C | 0.298 | -0.055 | 0.008 | 3.87E-11 | 771.74 |
| rs10234636 | T | C | 0.113 | 0.109 | 0.011 | 6.60E-21 | 1454.4 |
| rs1025497 | A | G | 0.373 | -0.049 | 0.007 | 2.89E-10 | 699.75 |
| rs10456100 | T | C | 0.282 | 0.061 | 0.008 | 7.75E-13 | 898.35 |
| rs10849061 | C | T | 0.486 | 0.063 | 0.007 | 9.36E-17 | 1196.8 |
| rs11153082 | G | A | 0.323 | 0.097 | 0.008 | 6.02E-34 | 2514.9 |
| rs11172113 | C | T | 0.428 | -0.117 | 0.007 | 1.15E-51 | 4055.9 |
| rs112255710 | T | C | 0.077 | -0.099 | 0.014 | 1.13E-11 | 849.65 |
| rs11624776 | C | A | 0.313 | -0.051 | 0.008 | 8.34E-10 | 686.79 |
| rs11657101 | A | G | 0.371 | 0.061 | 0.009 | 4.54E-11 | 1044.1 |
| rs11782673 | G | A | 0.164 | -0.057 | 0.010 | 3.46E-08 | 534.67 |
| rs12025158 | A | G | 0.351 | 0.058 | 0.007 | 1.69E-10 | 707.99 |
| rs13078967 | C | A | 0.026 | -0.162 | 0.025 | 1.11E-10 | 826.66 |
| rs17303101 | A | G | 0.292 | 0.069 | 0.008 | 2.37E-16 | 1185.7 |
| rs1925950 | G | A | 0.352 | 0.076 | 0.007 | 8.49E-22 | 1587.2 |
| rs2078371 | C | T | 0.118 | 0.131 | 0.011 | 5.34E-30 | 2135.6 |
| rs2274224 | C | G | 0.432 | -0.065 | 0.007 | 1.34E-17 | 1280.3 |
| rs2672592 | T | G | 0.362 | 0.044 | 0.007 | 1.97E-08 | 539.83 |
| rs28451064 | A | G | 0.133 | -0.065 | 0.011 | 2.26E-08 | 603.21 |
| rs34273564 | T | C | 0.482 | 0.042 | 0.007 | 3.06E-08 | 535.84 |
| rs42854 | G | C | 0.311 | 0.064 | 0.008 | 1.63E-15 | 1070.8 |
| rs4910165 | C | G | 0.322 | -0.066 | 0.008 | 4.23E-16 | 1156.3 |
| rs6046147 | T | C | 0.248 | 0.063 | 0.008 | 5.61E-13 | 882.91 |
| rs6057599 | T | C | 0.339 | 0.045 | 0.008 | 3.20E-08 | 538.31 |
| rs6904682 | T | C | 0.445 | -0.045 | 0.007 | 3.52E-09 | 603.99 |
| rs72926788 | C | T | 0.037 | -0.130 | 0.023 | 2.02E-08 | 726.19 |
| rs7518255 | A | G | 0.218 | 0.121 | 0.009 | 1.66E-41 | 3039.7 |
| rs7640543 | A | G | 0.321 | 0.046 | 0.008 | 1.34E-08 | 555.70 |
| rs7684253 | C | T | 0.448 | -0.043 | 0.007 | 1.00E-08 | 571.54 |
| rs7757975 | T | G | 0.157 | 0.089 | 0.010 | 6.47E-18 | 1277.4 |
| rs8075138 | T | C | 0.397 | 0. 077 | 0.007 | 9.07E-10 | 659.09 |
| rs9349379 | G | A | 0.411 | -0.084 | 0.007 | 6.69E-26 | 2042.2 |
| rs950570 | T | C | 0.070 | 0.085 | 0.014 | 1.29E-08 | 561.19 |
| rs953588 | T | C | 0.376 | 0.053 | 0.007 | 7.38E-12 | 805.78 |

SNP: single nucleotide polymorphism; A1: effect allele; A2: other allele; EAF: effect allele frequency.

**Table S7.** The members of the International Headache Genetics Consortium (IHGC).

| FirstName | MiddleName | LastName | Email | Department | Division | Institute | City | State | Country |
| --- | --- | --- | --- | --- | --- | --- | --- | --- | --- |
| Verneri |  | Anttila | verneri.anttila@gmail.com | Analytical and Translational Genetics Unit | Department of Medicine | Massachusetts General Hospital and Harvard Medical School | Boston | Massachusetts | USA |
| Verneri |  | Anttila |  | Program in Medical and Population Genetics |  | Broad Institute of MIT and Harvard | Cambridge | Massachusetts | USA |
| Verneri |  | Anttila |  | Stanley Center for Psychiatric Research |  | Broad Institute of MIT and Harvard | Cambridge | Massachusetts | USA |
| Ville |  | Artto | ville.artto@hus.fi | Department of Neurology |  | Helsinki University Central Hospital | Helsinki |  | Finland |
| Andrea | C | Belin | Andrea.Carmine.Belin@ki.se | Department of Neuroscience |  | Karolinska Institutet | Stockholm |  | Sweden |
| Anna |  | Bjornsdottir | abjorns@gmail.com | Neurology private practice |  | Laeknasetrid | Reykjavik |  | Iceland |
| Gyda |  | Bjornsdottir | gyda.bjornsdottir@decode.is |  |  | deCODE genetics/Amgen Inc. | Reykjavik |  | Iceland |
| Dorret | I | Boomsma | DI.Boomsma@psy.vu.nl | Netherlands Twin Register | Department of Biological Psychology | Vrije Universiteit | Amsterdam |  | the Netherlands |
| Sigrid |  | Børte | sigrid.borte@gmail.com | K.G. Jebsen Center for Genetic Epidemiology | Department of Public Health and Nursing, Faculty of Medicine and Health Sciences | Norwegian University of Science and Technology | Trondheim |  | Norway |
| Sigrid |  | Børte |  | Institute of Clinical Medicine | Faculty of Medicine | University of Oslo | Oslo |  | Norway |
| Sigrid |  | Børte |  | Research and Communication Unit for Musculoskeletal Health | Department of Research, Innovation and Education, Division of Clinical Neuroscience | Oslo University Hospital | Oslo |  | Norway |
| Mona | A | Chalmer | mona.ameri.chalmer@regionh.dk | Danish Headache Center | Department of Neurology | Copenhagen University Hospital | Copenhagen |  | Denmark |
| Daniel | I | Chasman | dchasman@research.bwh.harvard.edu | Department of Medicine | Division of Preventive Medicine | Brigham and Women's Hospital | Boston | Massachusetts | USA |
| Daniel | I | Chasman |  |  |  | Harvard Medical School | Boston | Massachusetts | USA |
| Bru |  | Cormand | bcormand@gmail.com | Department of Genetics | Spain Centre for Biomedical Network Research on Rare Diseases | University of Barcelona | Barcelona |  | Spain |
| Ester |  | Cuenca-Leon | estercuenca@gmail.com | Pediatric Neurology Research Group |  | Vall d'Hebron Research Institute | Barcelona |  | Spain |
| George |  | Davey-Smith | KZ.Davey-Smith@bristol.ac.uk | University of Bristol/Medical Research Council Integrative Epidemiology Unit |  | University of Bristol | Bristol |  | UK |
| Irene |  | de Boer | I.de_Boer.Neur@lumc.nl | Department of Neurology |  | Leiden University Medical Centre | Leiden |  | the Netherlands |
| Martin |  | Dichgans | Martin.Dichgans@med.uni-muenchen.de | Institute for Stroke and Dementia Research | University Hospital | LMU Munich | Munich |  | Germany |
| Martin |  | Dichgans |  |  |  | Munich Cluster for Systems Neurology | Munich |  | Germany |
| Tonu |  | Esko | tonu.esko@gmail.com | Estonian Biobank Registry | the Estonian Genome Center | University of Tartu | Tartu |  | Estonia |
| Tobias |  | Freilinger | tobias.kurth@charite.de | Department of Neurology |  | Klinikum Passau | Passau |  | Germany |
| Tobias |  | Freilinger |  | Department of Neurology and Epileptology | Hertie Institute for Clinical Brain Research | University of Tuebingen | Tuebingen |  | Germany |
| Padhraig |  | Gormley | padgorm@gmail.com |  |  | GSK Inc. | Cambridge | Massachusetts | USA |
| Lyn | R | Griffiths | lyn.griffiths@qut.edu.au |  | Centre for Genomics and Personalised Health | Queensland University of Technology | Brisbane | Queensland | Australia |
| Eija |  | Hämäläinen | eija.i.hamalainen@helsinki.fi | Institute for Molecular Medicine Finland | Helsinki Institute of Life Science | University of Helsinki | Helsinki |  | Finland |
| Thomas | F | Hansen | thomas.hansen@regionh.dk | Danish Headache Center | Department of Neurology | Copenhagen University Hospital | Copenhagen |  | Denmark |
| Thomas | F | Hansen |  |  | Novo Nordic Foundation Center for Protein Research | Copenhagen University | Copenhagen |  | Denmark |
| Aster | VE | Harder | A.V.E.Harder@lumc.nl | Department of Neurology |  | Leiden University Medical Centre | Leiden |  | the Netherlands |
| Aster | VE | Harder |  | Department of Human Genetics |  | Leiden University Medical Centre | Leiden |  | the Netherlands |
| Heidi |  | Hautakangas | heidi.hautakangas@helsinki.fi | Institute for Molecular Medicine Finland | Helsinki Institute of Life Science | University of Helsinki | Helsinki |  | Finland |
| Marjo |  | Hiekkala | marjo.hiekkala@helsinki.fi |  |  | Folkhälsan Research Center | Helsinki |  | Finland |
| Maria | G | Hrafnsdottir | mariahra@landspitali.is |  |  | Landspitali University Hospital | Reykjavik |  | Iceland |
| M. Arfan |  | Ikram | m.a.ikram@erasmusmc.nl | Department of Epidemiology |  | Erasmus University Medical Center | Rotterdam |  | the Netherlands |
| Marjo-Riitta |  | Järvelin | m.jarvelin@imperial.ac.uk | Department of Epidemiology and Biostatistics | MRC-PHE Centre for Environment and Health, School of Public Health | Imperial College London | London |  | UK |
| Marjo-Riitta |  | Järvelin |  | Center for Life Course Health Research | Faculty of Medicine | University of Oulu | Oulu |  | Finland |
| Marjo-Riitta |  | Järvelin |  |  | Unit of Primary Health Care | Oulu University Hospital, OYS | Oulu |  | Finland |
| Marjo-Riitta |  | Järvelin |  | Department of Life Sciences | College of Health and Life Sciences | Brunel University London | London |  | UK |
| Risto |  | Kajanne | risto.kajanne@helsinki.fi | Institute for Molecular Medicine Finland | Helsinki Institute of Life Science | University of Helsinki | Helsinki |  | Finland |
| Mikko |  | Kallela | mikko.kallela@pp.fimnet.fi | Department of Neurology |  | Helsinki University Central Hospital | Helsinki |  | Finland |
| Jaakko |  | Kaprio | jaakko.kaprio@helsinki.fi | Institute for Molecular Medicine Finland | Helsinki Institute of Life Science | University of Helsinki | Helsinki |  | Finland |
| Mari |  | Kaunisto | mari.kaunisto@helsinki.fi |  |  | Folkhälsan Research Center | Helsinki |  | Finland |
| Lisette | JA | Kogelman | lisette.kogelman@regionh.dk | Danish Headache Center | Department of Neurology | Copenhagen University Hospital | Copenhagen |  | Denmark |
| Espen | S | Kristoffersen | e.s.kristoffersen@medisin.uio.no | Research and Communication Unit for Musculoskeletal Health, Department of Research, Innovation and Education | Division of Clinical Neuroscience | Akershus University Hospital and University of Oslo | Oslo |  | Norway |
| Espen | S | Kristoffersen |  | Department of General Practice | Institute of Health and Society | University of Oslo | Oslo |  | Norway |
| Espen | S | Kristoffersen |  | Department of Neurology |  | Akershus University Hospital | Lørenskog |  | Norway |
| Christian |  | Kubisch | c.kubisch@uke.de |  | Institute of Human Genetics | University Medical Center Hamburg-Eppendorf | Hamburg |  | Germany |
| Mitja |  | Kurki | mitja.kurki@gmail.com | Psychiatric and Neurodevelopmental Genetics Unit | Department of Medicine | Massachusetts General Hospital | Boston | Massachusetts | USA |
| Tobias |  | Kurth | tobias.kurth@charite.de |  |  | Institute of Public Health | Charité – Universitätsmedizin |  | Berlin |
| Lenore |  | Launer | LaunerL@nia.nih.gov | Laboratory of Epidemiology and Population Sciences | Intramural Research Program | National Institute on Aging | Bethesda | Maryland | USA |
| Terho |  | Lehtimäki | terho.lehtimaki@tuni.fi | Department of Clinical Chemistry, Fimlab Laboratories, and Finnish Cardiovascular Research Center - Tampere | Faculty of Medicine and Health Technology | Tampere University | Tampere |  | Finland |
| Davor |  | Lessel | d.lessel@uke.de |  | Institute of Human Genetics | University Medical Center Hamburg-Eppendorf | Hamburg |  | Germany |
| Lannie |  | Ligthart | RSL.Ligthart@psy.vu.nl | Netherlands Twin Register | Department of Biological Psychology | Vrije Universiteit | Amsterdam |  | the Netherlands |
| Sigurdur | H | Magnusson | sigurdur.magnusson@decode.is |  |  | deCODE genetics/Amgen Inc. | Reykjavik |  | Iceland |
| Rainer |  | Malik | rainer.malik@med.uni-muenchen.de | Institute for Stroke and Dementia Research | University Hospital | LMU Munich | Munich |  | Germany |
| Bertram |  | Müller-Myhsok | bmm@mpipsykl.mpg.de |  |  | Max Planck Institute of Psychiatry | Munich |  | Germany |
| Carrie |  | Northover | cnorthover@23andme.com |  |  | 23&Me Inc. | Mountain View | California | USA |
| Dale | R | Nyholt | d.nyholt@qut.edu.au | School of Biomedical Sciences, Faculty of Health | Centre for Genomics and Personalised Health, Centre for Data Science | Queensland University of Technology | Brisbane | Queensland | Australia |
| Jes |  | Olesen | jes.olesen@regionh.dk | Danish Headache Center | Department of Neurology | Copenhagen University Hospital | Copenhagen |  | Denmark |
| Aarno |  | Palotie | aarno.palotie@helsinki.fi | Institute for Molecular Medicine Finland | Helsinki Institute of Life Science | University of Helsinki | Helsinki |  | Finland |
| Aarno |  | Palotie |  |  |  | University of Helsinki | Helsinki |  | Finland |
| Priit |  | Palta | priit.palta@helsinki.fi | Institute for Molecular Medicine Finland | Helsinki Institute of Life Science | University of Helsinki | Helsinki |  | Finland |
| Linda | M | Pedersen | lindampedersen@hotmail.com | Department of Research, Innovation and Education | Division of Clinical Neuroscience | Oslo University Hospital | Oslo |  | Norway |
| Nancy |  | Pedersen | nancy.pedersen@ki.se | Department of Medical Epidemiology and Biostatistics |  | Karolinska Institutet | Stockholm |  | Sweden |
| Matti |  | Pirinen | matti.pirinen@helsinki.fi | Institute for Molecular Medicine Finland | Helsinki Institute of Life Science | University of Helsinki | Helsinki |  | Finland |
| Matti |  | Pirinen |  | Department of Mathematics and Statistics |  | University of Helsinki | Helsinki |  | Finland |
| Matti |  | Pirinen |  | Department of Public Health |  | University of Helsinki | Helsinki |  | Finland |
| Danielle |  | Posthuma | d.posthuma@vu.nl | Department of Complex Trait Genetics | Center for Neurogenomics and Cognitive Research | Neuroscience Campus Amsterdam, VU University | Amsterdam |  | The Netherlands |
| Patricia |  | Pozo-Rosich | ppozo@vhebron.net | Headache Unit | Neurology Department | Vall d'Hebron University Hospital | Barcelona |  | Spain |
| Alice |  | Pressman | pressmar@sutterhealth.org |  |  | Sutter Health | Sacramento | California | USA |
| Olli |  | Raitakari | olli.raitakari@utu.fi | Centre for Population Health Research | University of Turku | Turku University Hospital | Turku |  | Finland |
| Olli |  | Raitakari |  | Research Centre of Applied and Preventive Cardiovascular Medicine |  | University of Turku | Turku |  | Finland |
| Olli |  | Raitakari |  | Department of Clinical Physiology and Nuclear Medicine |  | Turku University Hospital | Turku |  | Finland |
| Caroline |  | Ran | Caroline.Ran@ki.se | Department of Neuroscience |  | Karolinska Institutet | Stockholm |  | Sweden |
| Gudrun | R | Sigurdardottir | gudrun@setrid.is | Neurology private practice |  | Laeknasetrid | Reykjavik |  | Iceland |
| Hreinn |  | Stefansson | hreinn.stefansson@decode.is |  |  | deCODE genetics/Amgen Inc. | Reykjavik |  | Iceland |
| Kari |  | Stefansson | kari.stefansson@decode.is |  |  | deCODE genetics/Amgen Inc. | Reykjavik |  | Iceland |
| Olafur | A | Sveinsson | olafur@setrid.is |  |  | Landspitali University Hospital | Reykjavik |  | Iceland |
| Gisela | M | Terwindt | G.M.Terwindt@lumc.nl | Department of Neurology |  | Leiden University Medical Centre | Leiden |  | the Netherlands |
| Thorgeir | E | Thorgeirsson | thorgeir.thorgeirsson@decode.is |  |  | deCODE genetics/Amgen Inc. | Reykjavik |  | Iceland |
| Arn | MJM | van den Maagdenberg | A.M.J.M.van_den_Maagdenberg@lumc.nl | Department of Neurology |  | Leiden University Medical Centre | Leiden |  | the Netherlands |
| Arn | MJM | van den Maagdenberg |  | Department of Human Genetics |  | Leiden University Medical Centre | Leiden |  | the Netherlands |
| Cornelia |  | van Duijn | c.vanduijn@erasmusmc.nl | Department of Epidemiology |  | Erasmus University Medical Centre | Rotterdam |  | the Netherlands |
| Maija |  | Wessman | maija.wessman@helsinki.fi |  |  | Folkhälsan Research Center | Helsinki |  | Finland |
| Maija |  | Wessman |  | Institute for Molecular Medicine Finland | Helsinki Institute of Life Science | University of Helsinki | Helsinki |  | Finland |
| Bendik | S | Winsvold | bendik.s.winsvold@gmail.com | Department of Research, Innovation and Education | Division of Clinical Neuroscience | Oslo University Hospital | Oslo |  | Norway |
| Bendik | S | Winsvold |  | K.G. Jebsen Center for Genetic Epidemiology | Department of Public Health and Nursing, Faculty of Medicine and Health Sciences | Norwegian University of Science and Technology | Trondheim |  | Norway |
| Bendik | S | Winsvold |  | Department of Neurology |  | Oslo University Hospital | Oslo |  | Norway |
| John-Anker |  | Zwart | j.a.zwart@medisin.uio.no | Department of Research, Innovation and Education | Division of Clinical Neuroscience | Oslo University Hospital | Oslo |  | Norway |
| John-Anker |  | Zwart |  | K.G. Jebsen Center for Genetic Epidemiology | Department of Public Health and Nursing, Faculty of Medicine and Health Sciences | Norwegian University of Science and Technology | Trondheim |  | Norway |
| John-Anker |  | Zwart |  | Institute of Clinical Medicine | Faculty of Medicine | University of Oslo | Oslo |  | Norway |

**1.1 Supplementary Figures**

**
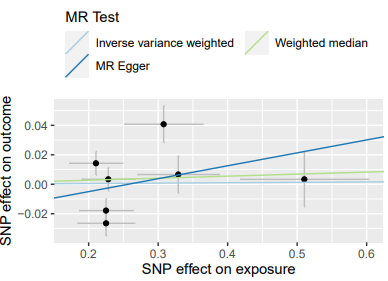
**

**Figure S1.** Scatter plot of “average acceleration” physical activity on migraine risk outcomes.

**
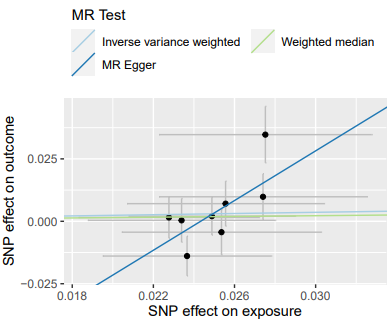
**

**Figure S2.** Scatter plot of “acceleration fraction > 425 mg” physical activity on migraine risk outcomes.

**
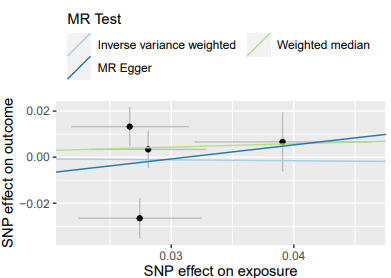
**

**Figure S3.** Scatter plot of overall physical activity on migraine risk outcomes.


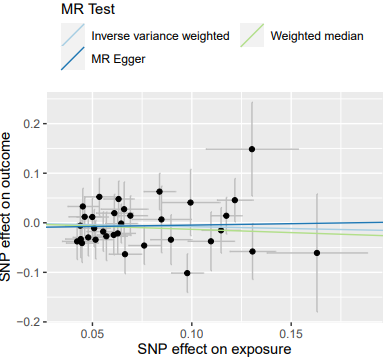


**Figure S4.** Scatter plot of migraine on “average acceleration” physical activity outcomes.


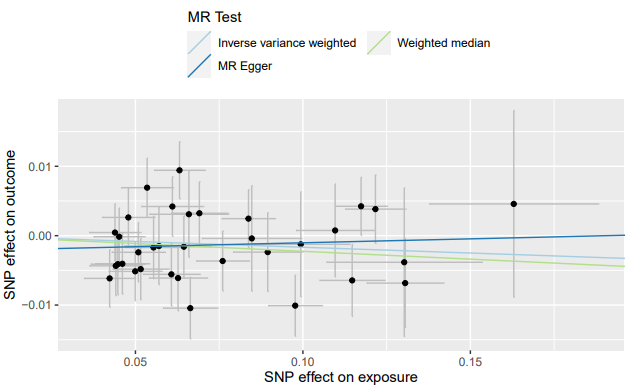


**Figure S5.** Scatter plot of migraine on “acceleration fraction >425 mg” physical activity outcomes.


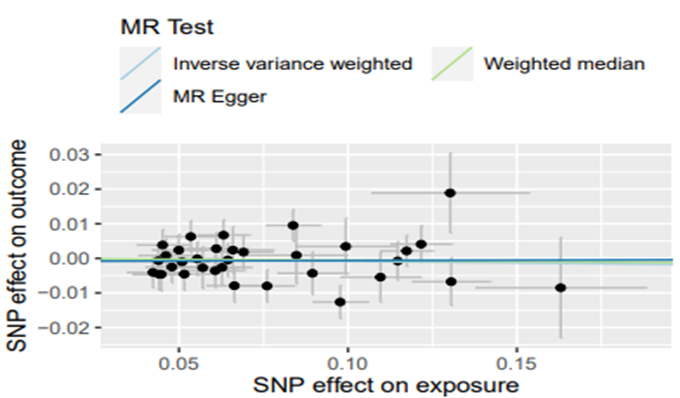


**Figure S6.** Scatter plot of migraine on overall physical activity outcomes.


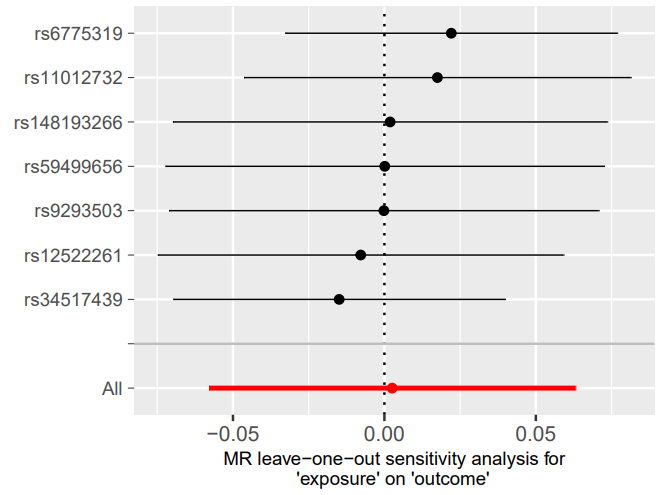


**Figure S7.** Leave−one−out sensitivity analysis between “average acceleration” physical activity and migraine (forward Mendelian randomization).


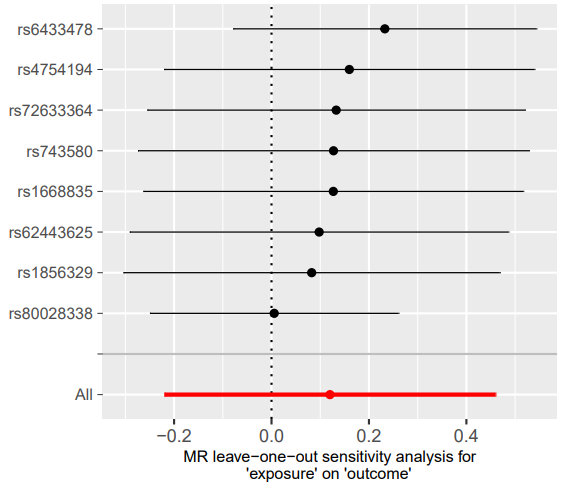


**Figure S8.** Leave−one−out sensitivity analysis between “acceleration fraction > 425 mg” physical activity and migraine (forward Mendelian randomization).


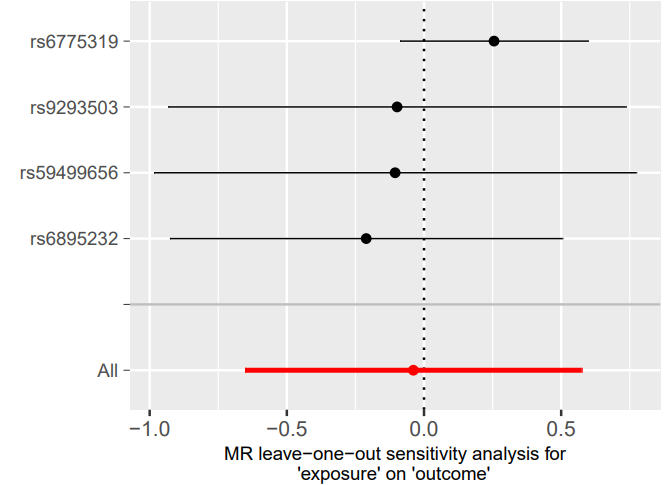


**Figure S9.** Leave−one−out sensitivity analysis between overall physical activity and migraine (forward Mendelian randomization).


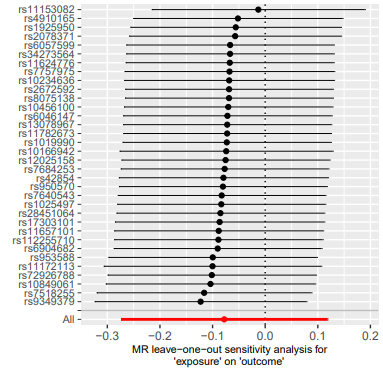


**Figure S10.** Leave−one−out sensitivity analysis between migraine and “average acceleration” physical activity (inverse Mendelian randomization).


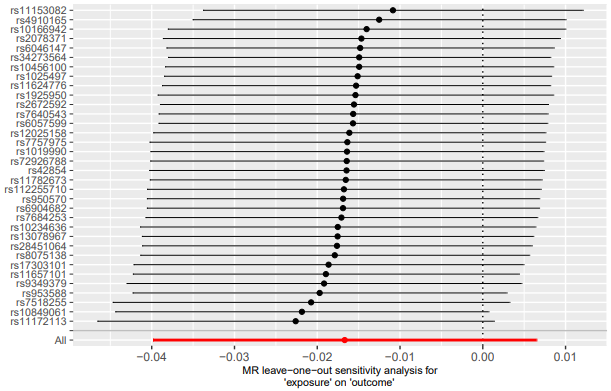


**Figure S11.** Leave−one−out sensitivity analysis between migraine and “acceleration fraction > 425 mg” physical activity (inverse Mendelian randomization).


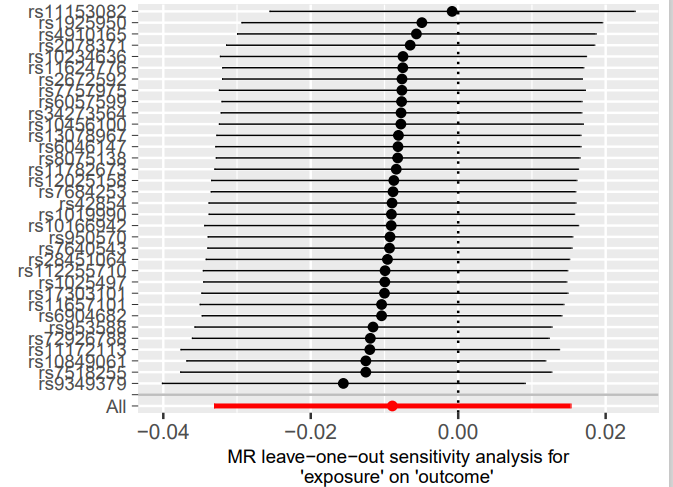


**Figure S12.** Leave−one−out sensitivity analysis between migraine and overall physical activity (inverse Mendelian randomization

**Figure S13.** Estimation of MR effects with removal of anomalous instrumental variables. *Abbreviations*: IV, Instrument variables; IVW, inverse variance-weighted.


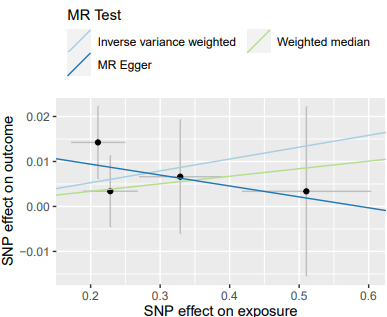


**Figure S14.** Scatter plot of “average acceleration” physical activity on migraine risk after removing abnormal SNPs.


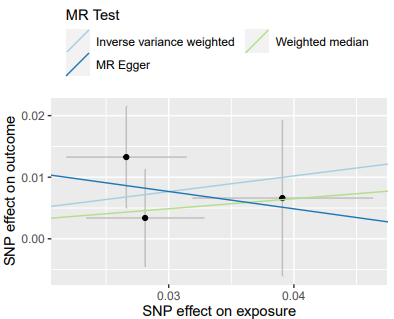


**Figure S15.** Scatter plot of overall physical activity on migraine risk outcome after removing abnormal SNPs.


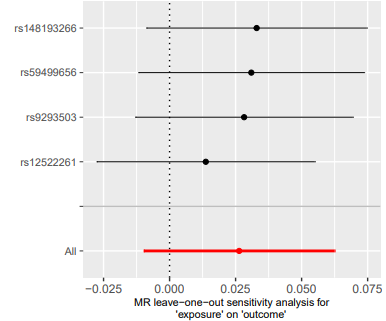


**Figure S16.** Leave-one-out analysis results of “average acceleration” physical activity on migraine risk after removing abnormal SNPs.


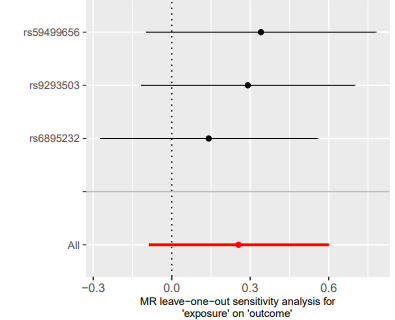


**Figure S17.** Leave-one-out analysis results of overall physical activity on migraine risk outcome after removing abnormal SNPs.


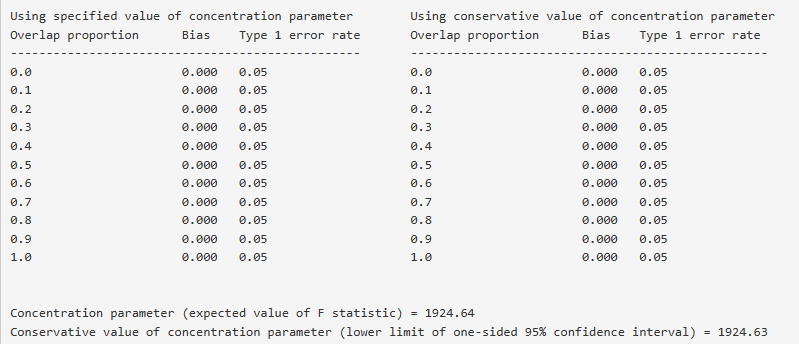


**Figure S18. “**Acceleration fraction > 425 mg” physical activity on migraine bias and type 1 error rates


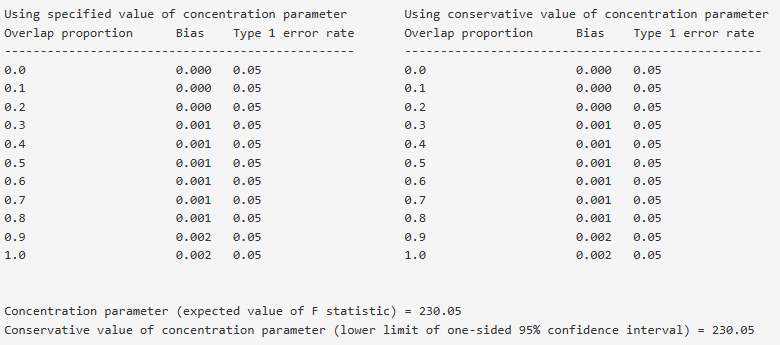


**Figure S19. “**Average acceleration” physical activity on migraine bias and type 1 error rates.


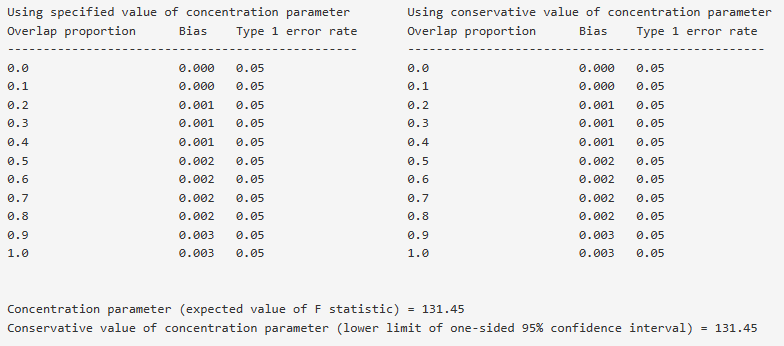


**Figure S20.** Overall physical activity on migraine bias and type 1 error rates.


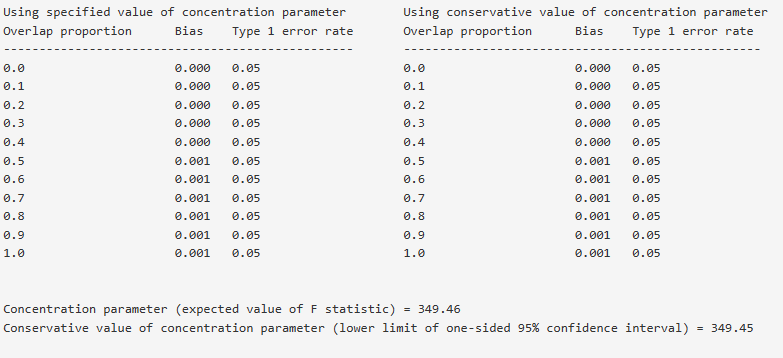


**Figure S21.** Migraine on “acceleration fraction >425 mg” physical activity bias and type 1 error rates.


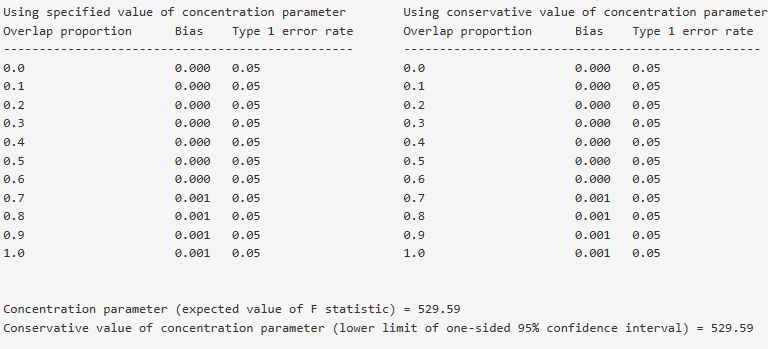


**Figure S22.** Migraine on “average acceleration” physical activity bias and type 1 error rates.


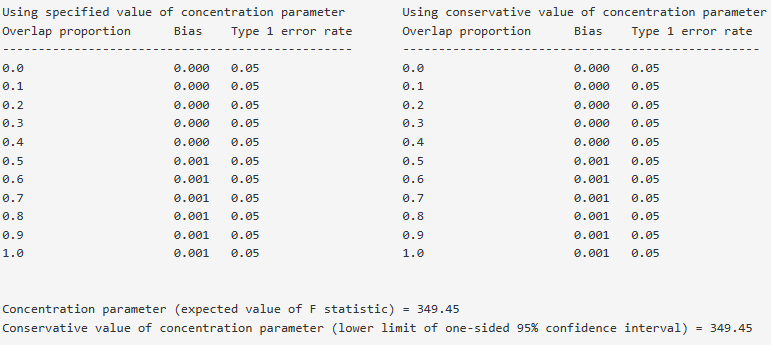


**Figure S23.** Migraine on overall physical activity bias and type 1 error rates.
